# Effects of Mediterranean Diet, Curcumin, and Resveratrol on Mild-to-Moderate Active Ulcerative Colitis: A Multicenter Randomized Clinical Trial

**DOI:** 10.3390/nu16101504

**Published:** 2024-05-16

**Authors:** Özge Erol Doğan, Kezban Esen Karaca Çelik, Murat Baş, Eyüp Hakan Alan, Yasir Furkan Çağın

**Affiliations:** 1 Department of Nutrition and Dietetics, Institute of Health Sciences, Acibadem Mehmet Ali Aydinlar University, Istanbul 34752, Turkey; 2 Department of Health Care Services, Vocational School of Health Services, Ardahan University, Ardahan 75002, Turkey; 3 Department of Nutrition and Dietetics, Faculty of Health Sciences, Acibadem Mehmet Ali Aydinlar University, Istanbul 34752, Turkey; 4 Department of Gastroenterology, Malatya Training and Research Hospital, Malatya 44330, Turkey; 5 Department of Gastroenterology, Faculty of Medicine, İnönü University, Malatya 44280, Turkey

**Keywords:** ulcerative colitis, Mediterranean diet, curcumin, resveratrol, supplementation, inflammatory bowel diseases

## Abstract

This study aimed to investigate the effects of the Mediterranean diet (MD), combined with curcumin and resveratrol supplementation, on disease activity, serum inflammatory markers, and quality of life in patients with mild-to-moderate active ulcerative colitis (UC). This study was designed as a prospective multicenter three-arm randomized controlled trial. Participants were randomized to the MD, MD + curcumin, and MD + resveratrol groups. All participants were placed on the MD for 8 weeks. The MD + curcumin group also received 1600 mg/day of curcumin supplementation, whereas the MD + resveratrol group received 500 mg/day of resveratrol supplementation for 8 weeks. Anthropometric measurements, Truelove–Witts Index, Short Form-36, Inflammatory Bowel Disease Questionnaire, Mediterranean Diet Adherence Scale (MEDAS), and laboratory tests were performed at baseline and postintervention. Within-group comparisons showed that MD, MD + curcumin, and MD + resveratrol interventions were effective in reducing disease activity and inflammation and improving quality of life in individuals with UC (*p* < 0.05). Between-group comparisons revealed no significant difference in all parameters except for the pain subparameter of SF-36 and the MEDAS score (*p* < 0.05). The MD is an effective and safe intervention to be used in clinical practice in individuals with UC.

## 1. Introduction

Ulcerative colitis (UC) is a chronic inflammatory condition that primarily affects the colon and rectal mucosa. It is characterized by recurrent episodes of symptoms, including diarrhea containing mucus and blood, rectal bleeding, abdominal pain, loss of appetite, weight loss, and elevated body temperature. UC is frequently accompanied by periods of remission, during which symptoms subside [1]. According to epidemiological data, the incidence of UC ranges from 1.2 to 20.3 cases per 100,000 individuals, and its prevalence ranges from 7.6 to 24.6 cases per 100,000 individuals [2,3,4]. In contemporary society, UC is a significant health issue that exhibits an escalating trend in terms of incidence and prevalence, which are influenced by factors including age, sex, race, geographical location, socioeconomic status, and dietary circumstances [5,6,7].

The etiology of UC is commonly linked to various factors, including infectious episodes, immunological deficiencies, mucin anomalies, dietary habits, and genetic and psychomotor disorders [8,9]. Dietary habits are a critical factor in regulating disease risk because they are essential for maintaining or achieving remission. The current literature states that vegetable- and fiber-rich diets can help decrease the risk of developing UC, whereas a diet high in omega-6 fatty acids, meat, refined sugar, and fast food can increase the risk of developing UC [10]. Recent studies have explored the effects of several dietary interventions, including a particular carbohydrate diet; a low fermentable oligosaccharides, disaccharides, monosaccharides, and polyols (FODMAP) diet; an anti-inflammatory diet; the Mediterranean diet (MD); and supplements, such as probiotics, symbiotics, polyphenol curcumin, and resveratrol, on disease management [11,12]. However, as stated in the ESPEN guidelines, there is insufficient evidence for specific dietary approaches in individuals with active disease [13].

The MD is a dietary pattern characterized by high consumption of plant foods, such as cereals, vegetables, and fruits, as well as olive oil, and small portions of dairy products, sweets, sugar, and meat products. It has anti-inflammatory effects and is beneficial for patients with inflammatory bowel disease (IBD) [14]. Studies have shown that stronger adherence to the MD in patients with UC can improve their quality of life and modulate disease activity [14,15,16]. A recent study that followed participants for 6 months reported that adopting the MD led to improved quality of life, increased remission rates, and favorable changes in anthropometric measurements in individuals with UC [17].

Curcumin is a pigment compound extracted from the rhizome of *Curcuma longa* by boiling and drying. Previous studies have demonstrated that curcumin inhibits the activity of two primary cytokines that control inflammatory responses: interleukin-1 and tumor necrosis factor alpha (TNF-α) [18]. In a recent meta-analysis conducted by Yin et al., data from 385 individuals with UC from six randomized controlled trials (RCTs) were analyzed. The findings indicated that curcumin supplementation was effective in achieving clinical remission; however, it did not have any impact on endoscopic remission [19].

Resveratrol, a biologically active natural polyphenol, is also a stilbenoid. In individuals with UC, the potential mechanisms of action of resveratrol include mitochondrial dysfunction prevention, inflammation modulation, and nuclear factor kappa B (NF-κB) production inhibition [20]. Although few studies have been conducted in human participants to investigate the effects of resveratrol on disease activity and severity in patients with UC, the results of these studies have suggested that resveratrol supplementation can improve symptoms, reduce serum proinflammatory cytokine levels, increase antioxidant capacity, and enhance the quality of life of patients [21,22].

To the best of our knowledge, studies exploring the impact of the MD, curcumin, and resveratrol on patients with UC are limited. In several of these studies, information regarding the dietary protocols implemented during the intervention period was insufficient. Consequently, whether the observed benefits are attributable to dietary supplements or dietary habits is unclear. The present study aimed to investigate the effects of the MD, in combination with curcumin and resveratrol supplementation, on disease activity, serum inflammatory markers, and quality of life in patients with mild-to-moderate active UC.

## 2. Materials and Methods

### 2.1. Participants

This study was conducted between February 2022 and January 2023 at the Gastroenterology Clinic of the Malatya Training and Research Hospital, Türkiye Health Sciences University, and the IBD Clinic of the Turgut Özal Medical Center, İnönü University. The following were the inclusion criteria: (1) diagnosis of mild-to-moderate active UC by a gastroenterologist, (2) age over 18 years, (3) use of mesalamine and/or azathioprine for medical treatment, and (4) written consent from the patients. The following were the exclusion criteria: (1) pregnancy; (2) breastfeeding; (3) chronic diseases (e.g., diabetes, hypothyroidism, hyperthyroidism, and liver, kidney, or cardiovascular system diseases); (4) use of anti-inflammatory and antibiotic medications; and (5) patients who declined voluntary participation and did not provide written consent.

G-Power 3.9.1.4 software (G-Power, Universität Düsseldorf, Germany) was used for a priori sample size analysis [23]. With a Type I error probability (α) of 0.05, a medium effect size (Cohen’s d = 0.50), and a desired power of 80%, the required sample size for the analysis was calculated to be 14 individuals per group, totaling 42 individuals (noncentrality parameter λ = 10.5/Critical F = 3.24). Considering the possibility of dropouts during the study, 48 individuals were included in the study, with 16 individuals in each group.

The Clinical Research Ethics Committee approved the study protocol (KN: 2021/24-30122021). The clinical trial registration number of this study was NCT05761327. Informed consent was obtained from all participants.

### 2.2. Study Design

**Figure 1:**
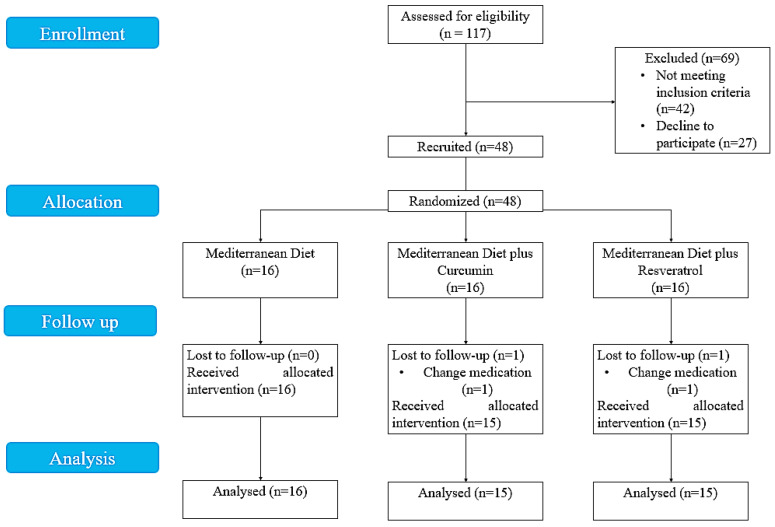
CONSORT diagram of the study.

This study was designed as a prospective multicenter three-arm RCT. Participants were randomly separated into the MD, MD + curcumin, and MD + resveratrol groups using Truelove–Witts Index-based block randomization with a 1:1:1 allocation ratio. All participants were evaluated at baseline and after 8 weeks of interventions. The CONSORT flow diagram is shown in Figure 1.

### 2.3. Interventions

**Figure 2:**
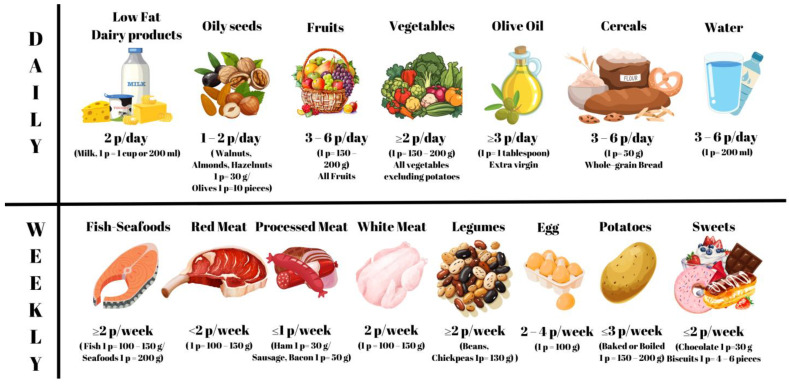
Recommended daily and weekly portions (p) and food types of the Mediterranean diet.

The participants in the control group were provided with the MD intervention for 8 weeks, with meetings held biweekly on a face-to-face basis with a dietitian. Dietary intervention was tailored to each individual’s physical activity levels (PALs), resting metabolic rate, and individual requirements following the ESPEN guidelines [13]. All participants received education on the MD pyramid, which included recommendations for the portions and frequencies of olive oil, nuts, olives, fish, yogurt, fruits, and vegetables, consistent with the principles of the MD, which typically comprise 40–45% carbohydrates, 15–20% protein, and 35–40% fat [24]. Participants were encouraged to consume at least three servings of vegetables per day, two to four servings of fruits per day, and fish twice a week (Figure 2). The other food groups were adjusted according to the MD pyramid and the energy needs of the participants [25]. The meal plan comprised three main meals and two to three snacks throughout the day.

Participants in the curcumin group adhered to the same dietary protocol as the control group and received 1600 mg/day of curcumin supplementation (VeNatura Curcumin Supplement; Vefa İlaç, Istanbul, Türkiye), divided into two capsules daily, morning and evening, for an 8-week period. The curcumin supplementation dosage was determined by previous studies [26].

Participants in the resveratrol group adhered to the same dietary protocol as the control group and received 500 mg/day of resveratrol supplementation (VeNatura Resveratrol Supplement; Vefa İlaç, Istanbul, Türkiye), divided into two capsules daily, morning and evening, for an 8-week period. The resveratrol supplementation dosage was determined by previous studies [20].

### 2.4. Data Collection

#### 2.4.1. Sociodemographic and General Information

At baseline, the participants’ sex, marital status, education level, employment status, smoking history, and dietary history related to UC were recorded.

#### 2.4.2. Anthropometric Measurements

Participants’ body weight (in kilograms), height (in centimeters), waist circumference (in centimeters), and hip circumference (in centimeters) were evaluated and documented at baseline and after intervention. The measurements were taken using standard equipment, including a SECA scale, stadiometer, and measuring tape (all from Seca, Hamburg, Germany), and were performed by the same researcher (OED). All measurements were taken in accordance with the International Standards for Anthropometric Assessment (ISAK) guidelines while wearing light-weight clothing and no shoes [27].

#### 2.4.3. Physical Activity

A 24-h physical activity diary was used to assess participants’ PALs. This diary categorized participants’ physical activities as “resting”, “very light activity”, “light activity”, “moderate activity”, and “vigorous activity” and calculated their total energy requirements accordingly. Subsequently, participants’ PALs were grouped into the following categories: “sedentary lifestyle (PAL = 1.00)”, “lightly active lifestyle (PAL = 1.40–1.69)”, “moderately active or active lifestyle (PAL = 1.70–1.99)”, and “vigorous or very active lifestyle (PAL = 2.00–2.40)” [28].

#### 2.4.4. Disease Activity

To evaluate the severity and disease activity of the participants, the Truelove–Witts Index was used [29]. This index assesses bowel movements (defecation frequency), presence of blood in stool, fever (pyrexia), elevated pulse rate, anemia, and erythrocyte sedimentation rate to categorize disease activity. The criteria for the Truelove–Witts Index are shown in Table 1.

#### 2.4.5. Inflammatory Biomarkers

Two gastroenterologists (EHA and YFC) recorded the complete blood count, C-reactive protein (CRP) levels, and erythrocyte sedimentation rate (ESR) at baseline and after intervention using the same laboratory equipment for evaluating serum inflammatory biomarkers.

#### 2.4.6. Food Intake Frequency and Dietary Records

Food consumption frequency was evaluated by inquiring about participants’ intake of fundamental food categories, including dairy and dairy products, meat, eggs, legumes and nuts, bread and grains, vegetables and fruits, and fats, over the past 3 months. The dietitian (OED) documented this information on the basis of options, such as “every meal, every day”, “1–2 times a week”, “3–4 times a week”, “5–6 times a week”, “once every 15 days”, “once a month”, “rarely”, and “never”.

Food consumption records were collected by asking participants to recall their intake for the past 24 h, covering two weekdays and one weekend day, and were documented by a dietician (OED). The information was subsequently analyzed using the BEBIS 9 (Nutrition Information System, İstanbul, Türkiye) program.

#### 2.4.7. Adherence to the MD

To assess participants’ commitment, the Mediterranean Diet Adherence Scale (MEDAS) was used, which comprises 14 items that are scored 0 or 1. In previous studies, two cutoff points were established for the MEDAS: seven or higher signifies acceptable adherence, whereas nine or higher represents strict adherence to the MD [30].

#### 2.4.8. Health-Related Quality of Life

To evaluate participants’ health-related quality of life, the Short Form-36 (SF-36) was used. The questionnaire comprises 36 items that are divided into eight subscales, including physical functioning (10 items), social functioning (2 items), physical role limitations (4 items), emotional role limitations (3 items), mental health (5 items), energy/vitality (4 items), pain (2 items), and general health perceptions (5 items). The scores for each subscale range from 0 to 100, with higher scores indicating better health-related quality of life [31].

#### 2.4.9. Disease-Related Quality of Life

To assess the overall disease-related quality of life of the patients, the Turkish adaptation of the Inflammatory Bowel Disease Questionnaire (IBDQ) was used. The IBDQ questionnaire comprises 32 items that are categorized into four distinct subscales, encompassing systemic symptoms (5 items), emotional function (12 items), social function (5 items), and bowel symptoms (10 items). The questionnaire uses a seven-point Likert scale system, with 1 point signifying the most severe impact and 7 points indicating no problem at all. The scores range from 32 to 224, with higher scores indicating a better quality of life [32].

### 2.5. Statistical Analysis

The 25th version of the Statistical Package for Social Sciences (SPSS Version 25.0, IBM, Armonk, NY, USA) for Windows was used for statistical analysis. The normal distribution of the data was evaluated using the Shapiro–Wilk test, histogram, kurtosis, and skewness. One-way analysis of variance (ANOVA) was used for between-group comparisons of parametric variables, and the Kruskal–Wallis test was used for nonparametric variables. To determine which group differences were significant, post hoc analysis with Bonferroni correction was conducted (*p* < 0.017). The independent samples *t*-test was used for pairwise comparisons of parametric variables, and the Mann–Whitney U test was used for nonparametric variables. For within-group comparisons, the paired samples *t*-test and Wilcoxon test were used for parametric and nonparametric variables, respectively. Chi-square McNemar tests were used to compare independent and dependent categorical variables, respectively. A statistical significance level of *p* < 0.05 was considered for all tests.

## 3. Results

This study involved 46 individuals, including 21 females and 25 males. The general characteristics and PALs of the participants are presented in Table 2. Among the participants, 68.8% were married, 35.4% had a high school diploma, 60% were employed, 79.2% were nonsmokers, and none had a specific dietary history related to the disease. Among the participants, 64.58%, 29.16%, and 6.25% had very light, light, and moderate physical activity, respectively.

The characteristics of the participants, including disease duration, age, anthropometric measurements, symptoms, and activity assessments, are summarized in Table 3. After statistical analyses, no significant differences were observed in the baseline and postintervention comparisons of these variables (*p* > 0.05). Within-group improvements in waist circumference, hip circumference, and bowel movements before and after the intervention showed statistical significance (*p* < 0.05).

The energy intake, macronutrient levels, and adherence to the MD of the participants are presented in Table 4. Between-group analysis showed no statistically significant differences in energy intake and macronutrient levels in the baseline and postintervention measurements. However, a significant difference in favor of the MD + C group was noted in the MEDAS scores after the intervention (*p* < 0.017). Within-group comparisons revealed a decrease in carbohydrate intake and an increase in protein and fat intake ratios in all groups. In addition, all participants demonstrated a statistically significant increase in their MEDAS scores (*p* < 0.05).

The hemograms and inflammatory biomarkers of the participants are presented in Table 5. Between-group analysis revealed no statistically significant differences in the hemogram and inflammatory biomarkers at baseline and postintervention (*p* > 0.05). Within-group comparisons showed that the MD, MD + C, and MD + R groups had a significant decrease in CRP and ESR levels. Additionally, significant improvements were observed in WBC, neutrophil, and neutrophil-to-lymphocyte ratio (NLR) levels only in the MD + R group. Furthermore, significant decreases in monocyte counts were detected in the MD + C and MD + R groups. Moreover, the MD group showed a significant increase in platelet distribution width (PDW; *p* < 0.05).

The health-related quality of life assessments of the participants are presented in Table 6. Between-group analysis showed no statistical differences in any parameter at baseline except for body pain (*p* > 0.05). The MD + R group reported lower levels of pain-related quality of life than the other groups (*p* < 0.05). Postintervention, the MD + C group showed a statistically significant improvement in social functioning (*p* < 0.05). Within-group comparisons revealed significant improvements in all parameters in the MD, MD + C, and MD + R groups (*p* < 0.05).

The disease-related quality of life assessments of the participants are presented in Table 7. Between-group analysis showed no statistical differences in any parameter at baseline (*p* > 0.05). The MD + C group showed better scores of the systemic symptoms subparameter postintervention (*p* < 0.05). Within-group comparisons revealed statistically significant improvements in all subparameters in the MD, MD + C, and MD + R groups (*p* < 0.05).

## 4. Discussion

The results of this study indicated that the MD was effective in achieving disease remission, regulating inflammatory markers, and enhancing the quality of life of individuals with mild-to-moderate active UC. Furthermore, this study is the first to examine the impact of curcumin and resveratrol supplements using a standardized and effective dietary model.

Anthropometric measurements play a crucial role in UC management by determining and monitoring nutritional status, predicting disease prognosis, and evaluating protein–energy malnutrition. In a study conducted by Lopes et al. (2022), participants showed lower body mass index (BMI), waist circumference, and body fat percentage than their healthy counterparts [33]. Another study revealed that BMI, body weight, and body fat were lower during the active phase of the disease than during the remission phase [34]. Chicco et al. reported that the MD was effective in reducing BMI, fat percentage, and waist circumference in patients with IBD [17]. A recent review demonstrated that the effects of curcumin on BMI reduction remain controversial; however, it may be effective in reducing waist circumference [35]. In a meta-analysis of 36 RCTs conducted by Tabrizi et al. in 2020, the effects of resveratrol on anthropometric measures, including body weight, BMI, waist circumference, and fat mass, were examined. The results showed that resveratrol supplementation was effective in increasing body weight and lean muscle mass in individuals with obesity. Furthermore, the use of resveratrol for periods exceeding 17 weeks was more effective than that for periods <8 or 8–16 weeks. The findings related to the daily resveratrol dose indicated that a daily intake of 200 mg or less of resveratrol was more effective than intakes of 200–500 and >500 mg [36]. The findings of this study align with those of previous research, demonstrating that MD, MD + C, and MD + R applications decrease waist and hip circumferences in patients. This may be attributed to a decrease in the fat ratio of the patients.

The active period of patients is a significant parameter that limits their PALs. A study using accelerometry reported that individuals with IBD showed less physical activity than their healthy peers [37]. A recent study conducted in 2019 showed that the PAL of individuals with UC decreased from the time of diagnosis [38]. Lyden et al. showed that the physical activity of individuals with UC during the active disease period tended to decrease [39]. Similarly, the results showed that a large proportion of patients did not have sufficient physical activity.

In 2023, a recent consensus report recommended the use of several different metrics for monitoring and standardizing UC disease activity. These metrics include the Simple Colitis Clinical Activity Index (SCCAI), Mayo Clinical Score, Ulcerative Colitis Disease Activity Index (UCDAI), and Truelove–Witts Index [40]. In contrast, SCCAI has been used in curcumin- and resveratrol-related studies [21,22,41,42]. The literature suggests that endoscopic and laboratory tests, which are accepted as the gold standard, should be applied together with clinical measurements to make more accurate inferences regarding disease severity and activity [43]. However, owing to the protocols, side effects, complications, discomfort, and cost, patients prefer endoscopic tests less [44,45]. A recent study recommended endoscopic examination at 6-month intervals for evaluating the effectiveness of a new treatment for mucosal healing [45]. Accordingly, owing to the limited study duration, the Truelove–Witts Index and laboratory tests, including ESR and CRP, were used to determine the severity and activity level of the disease. In future studies with long-term follow-up, we believe that endoscopic evaluation will contribute to mucosal healing evaluation.

A recent study demonstrated that the MD helps enhance disease activity and inflammatory markers in individuals with IBD [17]. According to a study of pediatric patients with IBD aged 12–18 years, the MD was successful in reducing CRP, calprotectin, and inflammatory cytokine levels and improving clinical scores [46]. It has been suggested that a daily intake of 1500 mg of curcumin can be effective in achieving remission in patients with UC with mild-to-moderately severe involvement [47]. An RCT conducted by Banerjee et al. in 2021 reported that patients with UC achieved clinical and endoscopic remission after receiving biologically enhanced curcumin supplements for 3 months [41]. Another study showed that 3 g of curcumin supplementation combined with mesalamine provided remission in individuals with mild-to-moderate UC with mild-to-moderate involvement and active disease [42]. Using a similar protocol, Hanai et al. demonstrated that daily 2 g of curcumin supplementation was effective in maintaining remission [48]. In contrast, Kedia, S. et al. showed that 150 mg of purified curcumin daily had no effect on UCDAI scores and remission in patients with mild-to-moderate UC [49]. Although few studies have examined the effects of resveratrol on disease severity and activity in patients with UC, a double-blind placebo-controlled pilot study by Samsamikor et al. in 2015 showed that 500 mg of resveratrol supplementation daily for 6 weeks was more effective in reducing disease severity and activity than the placebo [21]. In another study conducted by the same researcher in 2016, improvements in disease activity and severity were observed following resveratrol supplementation, which was examined for its oxidative/antioxidative effects in individuals with UC [22]. In the current study, no discernible difference was observed in the clinical index scores and inflammatory blood markers between the MD, MD + C, and MD + R groups in terms of disease activity and severity. This finding suggests that the primary therapeutic effect is attributable to the MD, implying that a dietary approach consistent with the MD model is a more effective treatment option than supplements for managing individuals with UC.

In patients with UC, adherence to the MD positively influences disease activity and quality of life [15]. Patients with UC with strict adherence to the MD appear to have lower disease activity scores and higher quality of life parameters than those with low adherence [50]. Moreover, the MD has been associated with reductions in inflammatory biomarker levels and changes in health-related microbial taxa and metabolites in patients [51]. The results showed that patients with UC in the MD, MD + C, and MD + R groups achieved high adherence to the MD.

Various blood parameters, including CRP, platelet count (PLC), mean platelet volume (MPV), NLR, platelet-to-lymphocyte ratio, hemoglobin, and ESR, are closely monitored for diagnosis, disease activity, and evaluation of treatment effectiveness in UC [52,53,54,55]. The latest American Gastroenterological Association guidelines have suggested that an increase in CRP levels above 5 mg/L can be associated with moderate-to-severe disease activity [56]. The current ECCO report states that this value is 10 mg/L [57]. Croft et al. observed that a 12 mg/L increase indicates active disease [58]. The UK disease surveillance report for IBD considers an ESR of <37 mm/h as an indicator of active severe colitis [59]. In this context, a decrease in CRP and ESR levels may be considered an indirect indicator of remission. A recent study showed that high adherence to the MD in pediatric patients was associated with decreased CRP levels [46]. According to a study examining the effects of curcumin supplementation in individuals with UC, 1500 mg of curcumin daily was effective in reducing serum hs-CRP levels [47]. Only one study examined the efficacy of resveratrol on hs-CRP levels. Based on the findings of this study, a 6-week 500 mg resveratrol supplementation led to a considerable decrease in the TNF-α, NF-κB, and hs-CRP levels of the patients [21]. Consistent with the existing literature, the current study demonstrated that the CRP and ESR levels decreased in the MD, MD + C, and MD + R groups. Of note, no significant difference was observed in the between-group comparisons postintervention, which may be attributed to the significant improvement observed in all the groups. This could be attributed to the anti-inflammatory and antioxidant properties of the MD.

Platelets, which are essential for maintaining homeostasis, secrete bioactive molecules that trigger proinflammatory processes in individuals with IBD [60]. Schneider et al. reported platelet abnormalities and thrombocytosis as significant findings in their study on UC [61]. Öztürk et al. asserted that platelet indices, including PLC, MPV, and PWD, serve as critical biomarkers for monitoring disease activity [62]. Furukawa et al. revealed that PLC was inversely proportional to mucosal healing in a cohort in Japan [63]. Moreover, Nakarai et al. indicated that PLC is a vital marker for predicting UC recurrence periods, with a PLC of more than 250.00 × 103 μL, which is a significant risk factor for disease activation [64]. In 2023, Gerçeker et al. demonstrated that PLT and PDW values are important biomarkers for determining mucosal healing, steroid resistance, and dependence in patients newly diagnosed with moderate-to-severe UC. In addition, the same study reported that PDW values were positively correlated with mucosal healing [65]. The results of this study are consistent with those of the existing literature on the indicators of active disease. The PLT values of patients in all three groups decreased postintervention compared with those in the preintervention period, whereas the PDW value increased. Although the differences in PLT and PDW did not reach statistical significance in all groups, these findings can be indirectly associated with a decrease in systemic inflammation, remission, and mucosal healing when evaluated holistically with other blood parameters. To increase the generalizability of the obtained information, future studies should examine fecal calprotectin, endoscopic evaluations, and proinflammatory serum cytokine levels.

In a 2022 retrospective cohort study by Mavroudis et al., the health-related quality of life data (SF-36) of 66 patients with UC were examined. The study reported that compared with the healthy population, the most affected SF-36 parameters were vitality, mental health, and emotional health-related parameters [66]. A recent study by Çelik et al. discovered that clinical activity was negatively correlated with the SF-36 subparameters of physical role difficulty, emotional role difficulty, vitality, mental health, and general health perception in individuals with IBD [15]. This study is the first RCT to evaluate the effects of an 8-week MD intervention on the health-related quality of life of patients with UC. The study, consistent with previous literature, observed that physical role difficulty, emotional role difficulty, vitality, and general health perception were the most affected SF-36 subdimensions by the intervention.

Few studies have examined the effects of curcumin supplementation on the health-related quality of life. A study conducted on 77 individuals with gastrointestinal symptoms noted that consuming 500 mg of curcumin daily for 8 weeks led to improvements in vitality, general health perception, pain, physical function, and physical role difficulty [67]. Moreover, another study reported that a daily intake of 500 mg of curcumin improved the quality of life of patients with colorectal cancer [68]. However, to the best of our knowledge, no RCTs have examined the impact of curcumin and resveratrol on the general health-related quality of life in individuals with UC. The results of the present study, which is the first of its kind in the literature, indicate that the use of curcumin and resveratrol supplements combined with the MD led to significant improvements in all SF-36 subparameters, suggesting a positive impact on the overall quality of life.

The literature recommends assessing disease-related quality of life in addition to health-related quality of life in individuals with UC [69]. A study conducted by Langhorst et al. in 2020 implemented a holistic approach that included body awareness, yoga, exercise, medication, nutrition, and personal habits training. The results of the study showed that the lifestyle changes implemented in the program were effective in improving emotional and systemic symptoms related to disease-related quality of life, particularly in individuals with active disease [70]. These findings align with previous research, such as a study by Chicco et al., which demonstrated that the MD can improve the IBDQ total score and increase disease-related quality of life in patients with both UC and CD [17]. The potential anti-inflammatory effects of the MD may contribute to improvements in the quality of life associated with intestinal and systemic symptoms, whereas secondary effects resulting from decreased disease activity may contribute to improvements in emotional and social functioning.

Sadeghi et al. demonstrated that curcumin supplementation improved disease-related quality of life in individuals with UC [47]. Only two studies have explored the effects of resveratrol supplementation on disease-related quality of life in individuals with UC. These studies by Samsamikor et al. showed that daily supplementation of 500 mg of resveratrol for 6 weeks improved the quality of life of patients with UC in the context of the IBDQ-9. Furthermore, these studies compared resveratrol supplementation with placebo intervention, and the results showed that resveratrol was superior to placebo in improving quality of life [21,22]. In one study, the effects of resveratrol differed according to sex. Specifically, in female mice, it resulted in adverse effects, whereas in male mice, it had no discernible effects [71]. The findings of this study align with the existing literature on disease-related quality of life. Between-group comparisons revealed a statistically significant difference in favor of the curcumin group compared with the resveratrol group only in the postintervention systemic symptom parameters. This may be because individuals taking resveratrol may have lower quality of life at baseline than those in the curcumin group. Additionally, the fact that patients in the curcumin group had higher compliance with the MD than those in the other groups may also explain this result. Also, we think that the gender-specific effects of resveratrol should be investigated in large samples in the future to better understand its effects.

The present study, which is the first of its kind in the literature, aimed to evaluate the impact of the MD, combined with supplementation with curcumin and resveratrol, on disease symptoms, inflammatory markers, and quality of life in individuals with UC from a multidisciplinary perspective. However, this study has some limitations. First, the absence of fecal calprotectin and proinflammatory cytokine levels and endoscopic imaging methods restricts the comprehensiveness of the obtained results. Moreover, this study was limited to individuals with mild-to-moderate active disease, which restricts the generalizability of the findings to individuals in remission or with severe active disease.

## 5. Conclusions

The results suggest that the MD is an effective and safe intervention to be used in clinical practice in individuals with UC. Furthermore, the results show that curcumin and resveratrol supplements do not produce a summation of effects when administered in addition to the dietary intervention. The authors also emphasize the need for additional research on Mediterranean diet patterns in patients with UC, particularly examining the effect of various macronutrient consumption rates.

## Figures and Tables

**Table 1:** Truelove–Witts Index.

Parameter	Mild	Moderate	Severe
Bowel movements	<4	4–5	≥6
Blood in the stool	No	Between mild and moderate	Yes
Fever (Pyrexia) ≥ 37.8 °C	No	No	Yes
Pulse ≥ 90/min	No	No	Yes
Anemia (Hemoglobin ≤ 10.5 g/dL)	No	No	Yes
ESR	≤30 mm/h	No	>30 mm/h

ESR: Erythrocyte sedimentation rate.

**Table 2:** General properties and physical activity levels of the participants at baseline.

	MD ^1^		MD + C ^2^		MD + R ^3^		Total	
	*n*	%	*n*	%	*n*	%	*n*	%
Sex								
Female	8	50	9	56.3	6	37.5	23	47.9
Male	8	50	7	43.8	10	62.5	25	52.1
Marital status								
Married	8	50	14	87.5	11	68.8	33	68.8
Single/divorced	8	50	2	12.5	5	31.3	15	31.3
Education level								
Primary school	3	18.8	6	37.5	3	18.8	12	25
Middle school	2	12.5	0	0	3	18.8	5	10.5
High school	6	37.5	7	43.8	4	25	17	35.4
Bachelor’s degree	3	18.8	3	18.8	4	25	10	20.8
Master of Science/Ph.D.	2	12.5	0	0	2	12.5	4	8.3
Employment status								
Yes	8	50	11	68.8	10	62.5	29	60.4
No	8	50	5	31.3	6	37.5	19	39.6
Smoking								
Yes	3	18.8	3	18.8	4	25	10	20.8
No	13	81.3	13	81.3	12	75	38	79.2
Dietary history								
Yes	0	0	0	0	0	0	0	0
No	16	100	16	100	16	100	48	100
PAL level								
<1.4	11	68.75	9	56.25	11	68.75	31	64.583
1.4–1.69	4	25	6	37.5	4	25	14	29.167
1.70–1.99	1	6.25	1	6.25	1	6.25	3	6.25
2.00–2.40	0	0	0	0	0	0	0	0

MD ^1^, Mediterranean diet; MD + C ^2^, Mediterranean diet + curcumin supplementation; MD + R ^3^, Mediterranean diet + resveratrol supplementation; *n*, count; %, frequency; Ph.D., doctor of philosophy.

**Table 3:** Age, anthropometric measurements, disease symptoms, and activity levels at baseline and after the interventions.

Parameters		MD ^1^	MD + C ^2^	MD + R ^3^	Between	
		X ± SD	X ± SD	X ± SD	*p*	T.V.
Duration (years)		4.41 ± 4.51	4.48 ± 3.69	5 ± 4.65	0.9	0.91 ^a^
Age (years)		40.12 ± 11.91	39.06 ± 11.16	38.5 ± 12.56	0.92	0.15 ^a^
Height (cm)		166.56 ± 8.17	165.75 ± 8.84	170.12 ± 8.94	0.33	0.21 ^a^
Weight (kg)	Pre	66.61 ± 11.16	73.84 ± 17.4	72.84 ± 15.38	0.49	1.42 ^a^
	Post	66.62 ± 10.34	73.39 ± 15.68	72.53 ± 14.91	0.37	1.95 ^a^
	Within	*p* = 0.65/Z = −0.45	*p* = 0.17/Z = −1.47	*p* = 0.68/Z = −0.41		
BMI (kg/m^2^)	Pre	23.94 ± 3.09	26.88 ± 6.08	25.07 ± 4.32	0.39	1.86 ^a^
	Post	23.96 ± 2.81	26.55 ± 5.4	24.71 ± 3.79	0.45	1.57 ^a^
	Within	*p* = 0.72/Z = −0.35	*p* = 0.14/Z = −1.47	*p* = 0.57/Z = −0.56		
WC (cm)	Pre	102.31 ± 7.18	106.47 ± 11.83	101.6 ± 7.52	0.54	1.23 ^a^
	Post	93.09 ± 10.13	96.19 ± 13.66	92.94 ± 11.24	0.71	0.67 ^a^
	Within	*p* = 0.001 */Z = −3.29	*p* = 0.001 */Z = −3.18	*p* = 0.008 */Z = −2.67		
HC (cm)	Pre	102.5 ± 6.96	104.93 ± 9.78	101.67 ± 7.13	0.71	0.69 ^a^
	Post	93.19 ± 9.19	95.01 ± 11.77	93.25 ± 10.91	0.76	0.53 ^a^
	Within	*p* = 0.001 */Z = −3.36	*p* = 0.001 */Z = −3.32	*p* = 0.007 */Z = −2.70		
WC/HC ratio	Pre	1.0 ± 0.02	1.01 ± 0.02	1.0 ± 0.02	0.25	7.334 ^a^
	Post	1.0 ± 0.01	1.01 ± 0.02	1.0 ± 0.01	0.24	2.8 ^a^
	Within	*p* = 1/Z = 0	*p* = 0.88/Z = −1.53	*p* = 0.92/Z = −0.11		
Bowel movements	Pre	3.56 ± 2.0	3.5 ± 1.51	3.38 ± 0.96	0.94	0.06 ^a^
	Post	1.31 ± 0.6	1.8 ± 1.15	1.67 ± 0.82	0.29	1.28 ^a^
	Within	*p* = 0.001 */t = 4.881	*p* = 0.001 */t = 4.063	*p* = 0.001 */t = 9.539		
Blood in the Stool		***n* (%)**	***n* (%)**	***n* (%)**	***p***	**T.V.**
Pre	Rare	13 (81.25)	12 (75)	14 (87.5)	0.66	0.82 ^c^
	Often	3 (18.75)	4 (25)	2 (12.5)		
Post	Rare	15 (93.75)	14 (93.33)	14 (93.33)	0.99	0.03 ^c^
	Often	1 (6.25)	1 (6.67)	1 (6.67)		
Within		*p* = 0.5 ^b^	*p* = 0.5 ^b^	*p* = 1 ^b^		
Truelove–Witts Index		***n* (%)**	***n* (%)**	***n* (%)**	***p***	**T.V.**
Pre	Mild	11 (68.75)	11 (68.75)	11 (68.75)	1	0 ^c^
	Moderate	5 (31.25)	5 (31.25)	5 (31.25)		
Post	Mild	15 (93.75)	13 (86.67)	13 (86.67)	0.76	0.54 ^c^
	Moderate	1 (6.25)	2 (13.33)	2 (13.33)		
Within		*p* = 0.13 ^b^	*p* = 0.5 ^b^	*p* = 0.25 ^b^		

MD ^1^, Mediterranean diet; MD + C ^2^: Mediterranean diet + curcumin supplementation; MD + R ^3^, Mediterranean diet + resveratrol supplementation; Between, comparisons between groups at baseline or after treatment; Within, comparisons between baseline and after treatment of each group; X, mean; SD, standard deviation; T.V., statistical test value; BMI, body mass index; WC, waist circumference; HC, hip circumference; Pre, baseline; Post, after treatment; t, paired samples *t*-test; Z, Wilcoxon; ^a^, Kruskall–Wallis; ^b^, McNemar; ^c^, Chi-square; *, *p* < 0.05.

**Table 4:** Energy intake, macronutrient levels, and adherence to the Mediterranean diet assessments of participants at baseline and after the interventions.

Parameters		MD ^1^	MD + C ^2^	MD + R ^3^	Between		Post Hoc
		X ± SD	X ± SD	X ± SD	*p*	T.V.	
Energy (kcal)	Pre	2166.12 ± 616.46	2459.74 ± 652.15	2531.58 ± 909.03	0.29	2.45 ^a^	
	Post	2128.88 ± 308.15	2139.05 ± 241.72	2205.88 ± 325.06	0.78	0.49 ^a^	
	Within	*p* = 0.92/Z = −0.1	*p* = 0.07/Z = −1.99	*p* = 0.33/Z = −0.97			
CHO (%)	Pre	51.31 ± 6.05	48.19 ± 5.77	48.25 ± 4.95	0.29	2.49 ^a^	
	Post	43.88 ± 0.72	43.67 ± 1.4	43.47 ± 0.99	0.48	1.48 ^a^	
	Within	*p* = 0.001 */Z = −3.47	*p* = 0.01 */Z = −2.59	*p* = 0.001 */Z = −2.82			
Protein (%)	Pre	14.94 ± 3.3	14.75 ± 1.67	14.41 ± 1.71	0.72	0.66 ^a^	
	Post	16.63 ± 1.75	16.6 ± 1.24	16.47 ± 1.13	0.91	0.19 ^a^	
	Within	*p* = 0.03 */Z = −2.15	*p* = 0.01 */Z = −2.74	*p* = 0.01 */Z = −2.55			
Fat (%)	Pre	33.91 ± 4.82	37.09 ± 5.8	37.22 ± 4.36	0.2	3.19 ^a^	
	Post	39.31 ± 1.3	39.6 ± 0.91	39.87 ± 0.92	0.48	1.47 ^a^	
	Within	*p* = 0.001 */Z = −3.1	*p* = 0.16/Z = −1.39	*p* = 0.04 */Z = −2.02			
MEDAS (Score)	Pre	2.88 ± 1.71	3.31 ± 1.7	3.19 ± 1.64	0.75	0.29 ^b^	
	Post	10.19 ± 1.22	11.27 ± 0.96	10.53 ± 1.19	0.03 *	3.64 ^b,x^	2 > 3 **
	Within	*p* = 0.001 */T= −15.49	*p* = 0.001 */Z = −14.72	*p* = 0.001 */Z = −16.45			

MEDAS, Mediterranean Diet Adherence Scale; MD ^1^, Mediterranean diet; MD + C ^2^, Mediterranean diet + curcumin supplementation; MD + R ^3^, Mediterranean diet + resveratrol supplementation; Between, comparisons between groups at baseline or after treatment; Within, comparisons between baseline and after treatment of each group; X, mean; SD, standard deviation; CHO, carbohydrate; Pre, baseline; Post, after treatment; T.V., statistical test value; ^a^, Kruskal–Wallis; ^b^, one-way ANOVA; Z, Wilcoxon; ^x^, Mann–Whitney U test; *, *p* < 0.05; Bonferroni correction **, *p*< 0.017, MD + C group showed more adherence to MD than MD + R group after treatment.

**Table 5:** Hemograms and inflammatory biomarkers of the patients at baseline and after interventions.

Parameters		MD ^1^	MD + C ^2^	MD + R ^3^	Between	
		X ± SD	X ± SD	X ± SD	*p*	T.V.
CRP (mg/dL)	Pre	1.58 ± 0.50	2.0 ± 2.41	1.31 ± 1.42	0.08	2.67 ^b^
	Post	0.32 ± 0.01	0.32 ± 0.01	0.36 ± 0.15	0.35	1.07 ^b^
	Within	*p* = 0.001 */T = 10.02	*p* = 0.01 */T = 2.83	*p* = 0.02 */T = 2.78		
ESR (mm/h)	Pre	10.94 ± 7.39	13.0 ± 7.57	14.81 ± 11.0	0.65	0.86 ^a^
	Post	6.81 ± 4.15	5.47 ± 3.62	5.0 ± 2.36	0.48	1.48 ^a^
	Within	*p* = 0.02 */Z = −2.32	*p* = 0.001 */T = 4.27	*p* = 0.001 */Z = −2.94		
WBC (10^3^/uL)	Pre	8.31 ± 2.58	8.65 ± 2.32	8.97 ± 3.03	0.89	0.23 ^a^
	Post	7.26 ± 1.61	8.05 ± 1.53	7.35 ± 2.72	0.2	3.23 ^a^
	Within	*p* = 0.13/Z = −1.5	*p* = 0.13/Z = −1.53	*p* = 0.03 */Z = −2.2		
HGB (g/dL)	Pre	13.86 ± 1.74	13.39 ± 2.06	13.14 ± 2.26	0.79	0.48 ^a^
	Post	14.19 ± 1.54	13.73 ± 1.81	13.91 ± 1.86	0.79	0.47 ^a^
	Within	*p* = 0.08/Z = −1.75	*p* = 0.69/Z = −0.4	*p* = 0.09/T = −1.82		
MCV (fL)	Pre	86.4 ± 7.69	85.0 ± 6.33	83.21 ± 5.85	0.18	3.48 ^a^
	Post	86.22 ± 7.49	85.1 ± 5.06	83.25 ± 5.42	0.21	3.16 ^a^
	Within	*p* = 0.6/Z = −0.52	*p* = 0.75/Z = −0.31	*p* = 0.87/Z = 0.16		
MPV (fL)	Pre	9.72 ± 1.0	9.6 ± 0.73	9.99 ± 0.88	0.58	1.09 ^a^
	Post	9.86 ± 0.69	9.65 ± 0.76	10.29 ± 1.2	0.24	2.88 ^a^
	Within	*p* = 0.29/Z = −1.06	*p* = 0.51/Z = −0.65	*p* = 0.17/Z = −1.38		
NE (10^3^/uL)	Pre	4.98 ± 1.89	5.31 ± 2.02	5.75 ± 2.72	0.8	0.45 ^a^
	Post	4.24 ± 1.29	4.6 ± 1.32	3.85 ± 1.75	0.19	3.3 ^a^
	Within	*p* = 0.23/Z = −1.19	*p* = 0.15/Z = −1.45	*p* = 0.01 */Z = −2.48		
LY (10^3^/uL)	Pre	2.36 ± 0.7	2.33 ± 0.56	2.24 ± 0.74	0.54	1.23 ^a^
	Post	2.3 ± 0.89	2.71 ± 0.76	2.45 ± 0.72	0.4	1.85 ^a^
	Within	*p* = 0.29/Z = −1.07	*p* = 0.15/Z = −1.45	*p* = 0.38/Z = −0.87		
NLR	Pre	2.2 ± 0.79	2.48 ± 1.32	2.74 ± 1.46	0.57	1.14 ^a^
	Post	2.3 ± 1.6	1.81 ± 0.69	1.6 ± 0.57	0.57	1.11 ^a^
	Within	*p* = 0.53/Z = −0.63	*p* = 0.23/Z = −1.19	*p* = 0.01 */Z = −2.48		
MO (10^3^/uL)	Pre	0.62 ± 0.26	0.76 ± 0.4	0.74 ± 0.25	0.15	3.77 ^a^
	Post	0.55 ± 0.23	0.65 ± 0.24	0.6 ± 0.39	0.15	3.84 ^a^
	Within	*p* = 0.12/Z = −1.56	*p* = 0.04 */Z = −2.07	*p* = 0.03 */Z = −2.12		
PLT (10^3^/uL)	Pre	342.75 ± 157.55	322.0 ± 71.23	330.19 ± 59.66	0.84	0.35 ^a^
	Post	327.44 ± 123.45	298.07 ± 64.3	308.0 ± 67.59	0.82	0.39 ^a^
	Within	*p* = 0.27/Z = −1.11	*p* = 0.16/Z = −1.39	*p* = 0.25/Z = −1.15		
PDW (fL)	Pre	11.02 ± 2.1	10.48 ± 1.46	11.27 ± 1.84	0.55	1.19 ^a^
	Post	11.59 ± 1.83	10.49 ± 1.58	12.21 ± 2.78	0.15	3.78 ^a^
	Within	*p* = 0.04 */Z = −2.05	*p* = 0.73/Z = −0.35	*p* = 0.08/Z = −1.77		

MD ^1^: Mediterranean diet; MD + C ^2^, Mediterranean diet + curcumin supplementation; MD + R ^3^, Mediterranean diet + resveratrol supplementation; Between, comparisons between groups at baseline or after treatment; Within, comparisons between baseline and after treatment of each group; X, mean; SD, standard deviation; CRP, C-reactive protein; ESR, erythrocyte sedimentation rate; WBC, white blood cell count; HGB, hemoglobin; MCV, mean corpuscular volume; MPV, mean platelet volume; NE, neutrophil; LY, lymphocyte; NLR, neutrophil-to-lymphocyte ratio; MO, monocyte; PLT, platelet; PDW, platelet distribution width; Pre, baseline; Post, after treatment; T.V., statistical test value; ^a^, Kruskal–Wallis; ^b^, one-way ANOVA; Z, Wilcoxon; T, paired samples *t*-test; *, *p* < 0.05.

**Table 6:** Investigation of the health-related quality of life.

SF-36 (Score)		MD ^1^	MD + C ^2^	MD + R ^3^	Between		Post Hoc
		X ± SD	X ± SD	X ± SD	*p*	T.V.	
PF	Pre	70.62 ± 32.4	68.12 ± 28.57	61.25 ± 26.04	0.37	1.97 ^a^	
	Post	81.88 ± 28.04	88.0 ± 16.78	73.44 ± 25.8	0.25	1.42 ^b^	
	Within	*p* = 0.04 */Z = −2.14	*p* = 0.01 */Z = −2.68	*p* = 0.01 */Z = −2.54			
RP	Pre	62.5 ± 39.79	50.0 ± 38.73	51.56 ± 40.28	0.63	0.47 ^b^	
	Post	93.75 ± 25.0	93.33 ± 25.82	81.67 ± 38.34	0.46	0.78 ^b^	
	Within	*p* = 0.001 */T = −3.37	*p* = 0.001 */T = −4.26	*p* = 0.01 */T = −3.16			
RE	Pre	62.5 ± 36.26	66.67 ± 34.43	45.83 ± 34.16	0.22	1.59 ^b^	
	Post	93.75 ± 25.0	91.11 ± 26.63	93.33 ± 25.82	0.95	0.05 ^b^	
	Within	*p* = 0.001 */T = −3.76	*p* = 0.01 */T = −1.79	*p* = 0.001 */T = −5.29			
VT	Pre	57.19 ± 23.45	49.69 ± 24.8	40.94 ± 22.82	0.14	3.95 ^a^	
	Post	66.25 ± 21.17	73.33 ± 19.33	63.0 ± 20.25	0.26	2.69 ^a^	
	Within	*p* = 0.04 */Z = −2.1	*p* = 0.001 */Z = −3.21	*p* = 0.001 */Z = −3.06			
MH	Pre	71.75 ± 17.49	71.0 ± 20.53	57.25 ± 21.3	0.09	4.83 ^a^	
	Post	79.5 ± 17.52	84.0 ± 12.92	73.6 ± 15.25	0.19	3.28 ^a^	
	Within	*p* = 0.001 */Z = −3.08	*p* = 0.001 */Z = −2.97	*p* = 0.001 */Z = −3.07			
SF	Pre	69.53 ± 35.05	64.84 ± 33.61	61.72 ± 25.19	0.56	1.16 ^a^	
	Post	85.16 ± 17.81	95.83 ± 16.14	90.0 ± 17.8	0.03*	7.02 ^a,x^	2 > 1 **^, c^
	Within	*p* = 0.01 */T = −3.02	*p* = 0.001 */T = −3.72	*p* = 0.001 */Z = −2.92			
BP	Pre	74.69 ± 29.75	57.34 ± 29.28	50.31 ± 23.18	0.03 *	7.28 ^a^	3 > 1 **^, d^
	Post	87.03 ± 16.1	91.17 ± 14.7	84.67 ± 17.45	0.44	1.64 ^b,x^	
	Within	*p* = 0.03 */T = −2.24	*p* = 0.001 */Z = −3.07	*p* = 0.001 */Z = −3.19			
GH	Pre	41.56 ± 20.31	39.06 ± 24.17	37.19 ± 12.38	0.73	0.63 ^a^	
	Post	53.44 ± 24.95	49.33 ± 24.49	54.33 ± 20.6	0.8	0.44 ^a^	
	Within	*p* = 0.01 */Z = −2.61	*p* = 0.02 */Z = −2.43	*p* = 0.001 */Z = −3.2			

SF-36, Short Form-36; MD ^1^, Mediterranean diet; MD + C ^2^, Mediterranean diet + curcumin supplementation; MD + R ^3^, Mediterranean diet + resveratrol supplementation; Between, comparisons between groups at baseline or after treatment; Within, comparisons between baseline and after treatment of each group; X, mean; SD, standard deviation; PF, physical functioning; RP, physical role functioning; RE, emotional role functioning; VT, vitality; MH, mental health; SF, social functioning; BP, body pain; GH, general health; Pre, baseline; Post, after treatment; T.V., statistical test value; ^a^, Kruskal–Wallis; ^b^, one-way ANOVA; Z, Wilcoxon; T, paired samples *t*-test; ^x^, Mann–Whitney U test; *, *p* < 0.05; Bonferroni correction, **, *p* < 0.017, ^c^ MD + R group showed more social functioning than MD group at after treatment, ^d^ MD + R group had more bodily pain than MD group at baseline.

**Table 7:** Investigation of the disease-related quality of life.

IBDQ (Score)		MD ^1^	MD + C ^2^	MD + R ^3^	Between		Post Hoc
		X ± SD	X ± SD	X ± SD	*p*	T.V.	
BS	Pre	49.0 ± 14.01	40.94 ± 14.12	44.44 ± 14.21	0.15	3.76 ^a^	
	Post	59.12 ± 8.16	61.87 ± 7.15	57.4 ± 7.19	0.22	3.03 ^a^	
	Within	*p* = 0.001 */Z = −3.52	*p* = 0.001 */Z = −3.35	*p* = 0.01 */Z = −2.56			
SS	Pre	20.69 ± 4.13	20.56 ± 5.42	17.06 ± 3.86	0.07	5.23 ^a^	
	Post	30.06 ± 3.23	31.47 ± 3.93	28.8 ± 3.67	0.04 *	5.97 ^a,x^	2 > 3 **^, c^
	Within	*p* = 0.001 */Z = −3.52	*p* = 0.001 */Z = −3.42	*p* = 0.001 */Z = −3.42			
EF	Pre	60.19 ± 11.25	53.06 ± 15.34	47.94 ± 13.85	0.06	5.92 ^a^	
	Post	70.5 ± 9.0	73.67 ± 11.16	67.2 ± 11.86	0.2	3.22 ^a^	
	Within	*p* = 0.001 */Z = −3.33	*p* = 0.001 */Z = −3.41	*p* = 0.001 */Z = −3.41			
SF	Pre	24.75 ± 8.84	22.44 ± 9.49	21.06 ± 6.42	0.32	2.27 ^a^	
	Post	32.0 ± 4.2	32.93 ± 4.86	31.33 ± 4.61	0.63	0.47 ^b^	
	Within	*p* = 0.001 */Z = −3.18	*p* = 0.001 */T = −4.95	*p* = 0.001 */Z = −3.41			
Total	Pre	154.56 ± 32.41	137.0 ± 38.61	130.5 ± 31.59	0.15	3.81 ^a^	
	Post	191.69 ± 21.57	199.93 ± 24.76	184.73 ± 24.09	0.06	5.52 ^a^	
	Within	*p* = 0.001 */Z = −3.47	*p* = 0.001 */Z = −3.41	*p* = 0.001 */Z = −3.41			

IBDQ, Inflammatory Bowel Disease Questionnaire; MD ^1^, Mediterranean diet; MD + C ^2^, Mediterranean diet + curcumin supplementation; MD + R ^3^, Mediterranean diet + resveratrol supplementation; Between, comparisons between groups at baseline or after treatment; Within, comparisons between baseline and after treatment of each group; X, mean; SD, standard deviation; BS, bowel symptoms; SS, systemic symptoms; EF, emotional functions; SF, social function; Pre, baseline; Post, after treatment; T.V., statistical test value; ^a^, Kruskal–Wallis; ^b^, one-way ANOVA; Z, Wilcoxon; T, paired samples *t*-test; ^x^, Mann–Whitney U test; *, *p* < 0.05; Bonferroni correction **, *p* < 0.017, ^c^, MD + C group had a better quality of life than MD + R in context of systemic symptoms at after treatment.

## Data Availability

The data obtained in this study are available from the corresponding author upon request.
